# Pediatric Flexible Endoscopic Evaluation of Swallowing: Critical Analysis of Implementation and Future Perspectives

**DOI:** 10.1007/s00455-021-10312-5

**Published:** 2021-04-28

**Authors:** Jana Zang, Julie Cläre Nienstedt, Jana-Christiane Koseki, Almut Nießen, Till Flügel, Susan Hyoungeun Kim, Christina Pflug

**Affiliations:** Department of Voice, Speech and Hearing Disorders, Center for Clinical Neurosciences, University Medical Center Hamburg‐Eppendorf, Hamburg, Germany

**Keywords:** Pediatric FEES, Deglutition disorder, Dysphagia assessment, Pediatric swallowing disorders

## Abstract

This study aimed to critically review pediatric swallowing assessment data to determine the future need for standardized procedures. A retrospective analysis of 152 swallowing examinations in 128 children aged 21 days to 18 years was performed. The children were presented at a university dysphagia center between January 2015 and June 2020 for flexible-endoscopic evaluation of swallowing (FEES). Descriptive analysis was conducted for the sample, swallowing pathologies, diagnosis, and missing values. Using binary logistic regression, the relationship between dysphagia and underlying diseases was investigated. The largest group with a common diagnosis in the cohort were children with genetic syndromes (*n* = 43). Sixty-nine children were diagnosed with dysphagia and 59 without dysphagia. The non-dysphagic group included 15 patients with a behavioral feeding disorder. The presence of an underlying disease significantly increased the chance of a swallowing problem (OR 13.08, 95% CI 3.66 to 46.65, *p* = .00). In particular, the categories *genetic syndrome* (OR 2.60, 95% CI 1.15 to 5.88) and *neurologic disorder* (OR 4.23, 95% CI 1.31 to 13.69) were associated with higher odds for dysphagia. All pediatric FEES were performed without complications, with a completion rate of 96.7%, and with a broad variability of implementation. Several charts lacked information concerning swallowing pathologies, though. Generally, a more standardized protocol and documentation for pediatric FEES is needed to enable better comparability of studies on epidemiology, assessment, and treatment outcomes in future.

**Supplementary Information:**

The online version contains supplementary material available at 10.1007/s00455-021-10312-5.

## Introduction

Despite an increasing prevalence of dysphagia [1–3] and a high demand for interdisciplinary diagnostics and therapy, pediatric patients still are underrepresented in dysphagia research. A further increase might be expected due to the higher chance of survival of very premature children [4] and of children with complex diseases who are at risk of dysphagia [5]. Besides, there is a lack of standardized diagnostic procedures both in clinical practice and in research [6]. No internationally accepted definition of pediatric swallowing disorders is available to date; most published studies are not based on the same definition or do not differentiate between behavioral feeding disorders and dysphagia [7]. This causes difficulties in comparison and replication, e.g., of epidemiological surveys [1, 8], and limitation of the informative content.

Swallowing disorders in children have a significant impact on health, cognitive development [9], and quality of life of the entire family [10, 11]. Due to these burdening consequences, the use of well-evaluated diagnostic instruments and clear criteria for early detection is mandatory to establish early and appropriate therapy [12–14].

So far, clinical swallowing evaluation (CSE) on its own cannot validly predict aspiration [12, 15]. Besides, currently, there are no valid clinical markers or predictors for oropharyngeal dysphagia with aspiration in children [16–20].

Descriptions of pediatric FEES routines were recently published by Miller, Schroeder, and Langmore [21] and Miller and Willging [22]. Modified procedures especially for breastfeeding [23, 24] or for the neonatal intensive care unit (NICU) [25, 26] have been tried and found to be safe. Objective methods for a transfer into a score such as the penetration-aspiration scale (PAS) according to Rosenbek [27] have not yet been validated for pediatric FEES, but are frequently in use.

This study aimed to systematically evaluate the pediatric swallowing diagnostics carried out at our university dysphagia center. The underlying hypothesis was that the lack of a standard protocol leads to gaps in documentation and thus poor comparability of findings. The results of this study are intended to serve as the fundament for subsequent development of standard pediatric FEES protocol and documentation.

## Methods

**Fig. 1:**
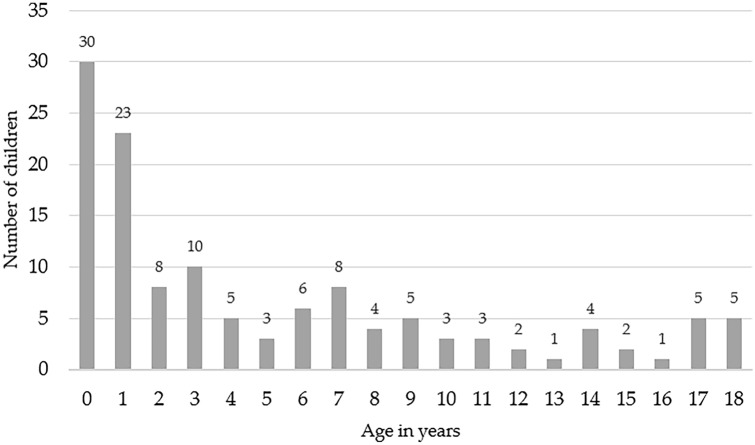
Age distribution (*N* = 128). Age in years (0–18) and the number of children

In this study, the electronic medical records of 152 swallowing examinations of 128 children aged 21 days to 18 years performed at a university dysphagia center between January 2015 and June 2020 were analyzed (see Table 1 and Fig. 1 for age distribution).

**Table 1:** Sample profile (*N* = 128)

Age in years^a^		Gender (%)		Tube feeding (%)		Ventilation (%)	
Mean ± SD	Range	Male	Female	PEG	NGT	LTV	NIV
5.5 ± 5.5	0.06–18.83	70 (54.7)	58 (45.3)	21 (16.4)	8 (6.3)	1 (0.8)	6 (4.7)

^a^In case of multiple examinations: age at first examination

*SD* standard deviation, *PEG* percutaneous endoscopic gastrostomy, *NGT* nasogastric tube, *LTV* long-term ventilation, *NIV* non-invasive ventilation at night

### Swallowing Examination

FEES was performed by experienced specialists in phoniatrics and otorhinolaryngology, using a 2.6 mm diameter high-definition rhino-laryngo-videoscope (ENF-V3, Olympus Medical Systems Corp., Tokyo, Japan), and accompanied by a speech-language pathologist (SLP) and a nurse. Age-appropriate dosage of nasal decongestant (Otriven, Xylometazoline hydrochloride: 0–2 years 0.25 mg; 2–6 years 0.5 mg; > 6 years: 1.0 mg) and topical viscous lidocaine (Xylocaine Gel 2%, Aspen Germany, 0.1 ml) were applied routinely. Nasogastric tubes were usually not removed, and pulse oximetry monitoring was only used for medically complex children. The young patients sat upright on the caregiver's lap, if possible, with the nurse stabilizing their head until the endoscope was passed through the nasal airway. Developmentally appropriate test boluses of different consistencies (e.g., fluid, thickened fluid, nectar thick or honey-thick, puree, solid) were administered by spoon, cup, bottle, or syringe in non-standardized bolus sizes. Mainly the children's preferred food was brought from home and lightly dyed with green food color ("Condi Light Green" E104 Quinoline Yellow + E132 Indigotine I, Schreiber-Essenzen GmbH & Co KG, Barsbüttel, Germany). Fluids or thin puree were thickened using modified corn starch (Thick & Easy, Fresenius Kabi, Germany). The procedure of FEES was carried out according to the FEES protocol of Langmore [28–30], albeit in non-standard modifications. The implementation depended on the examiner and the patient. Occasionally, only one consistency could be examined, and in some rare cases, the endoscopy was performed only immediately after oral intake of the bolus to check for residues and aspiration. The supplementary videos 1 to 4 give an impression of the examinations carried out on children of different ages and diagnoses.

### Statistical Analysis

Statistical analysis was performed using SPSS Statistic version 27 (IBM, USA). Descriptive analysis was conducted for the number of cases, the sample profile (age, gender, underlying disease, tube feeding, ventilation), functional swallowing pathologies (aspiration, laryngeal penetration, spillage, residue, delayed swallowing reflex, reduced laryngopharyngeal sensation), and the resulting description of the diagnosis. Binary logistic regression (forward, stepwise) was calculated for dichotomous variables (disease/no disease; dysphagia/no dysphagia). The level of significance was 0.05.

## Results

### Subject Characteristics

As summarized in Table 1, 45.3% of the 128 children were female and 54.7% male. There was a large number of children with genetic syndromes (see Table 2), including children with rare diseases such as Pompe disease (Glycogen storage disease type II) and spinal muscular atrophy (SMA) type I and II.

**Table 2:** Underlying diseases^a^ (*N* = 128)

	*n* (%)
None/unknown	25 (19.5)
Prematurity (GA^b^ ≤ 36 weeks)	14 (10.9)
Genetic syndrome^c^	43 (33.6)
Anatomical deviations	4 (3.1)
Neurologic disorder	18 (14.1)
Cardiorespiratory	8 (6.2)
Gastroenterological	7 (5.5)
Other	9 (7.0)

^a^Primary diagnosis

^b^Gestational age: average gestational age of children with the primary diagnosis of premature birth was 30.7 weeks (± 4.4; range 24–36)

^c^Genetic syndrome confirmed *n* = 30, suspected genetic syndrome, not yet confirmed *n* = 13

### Swallowing Examination

**Fig. 2:**
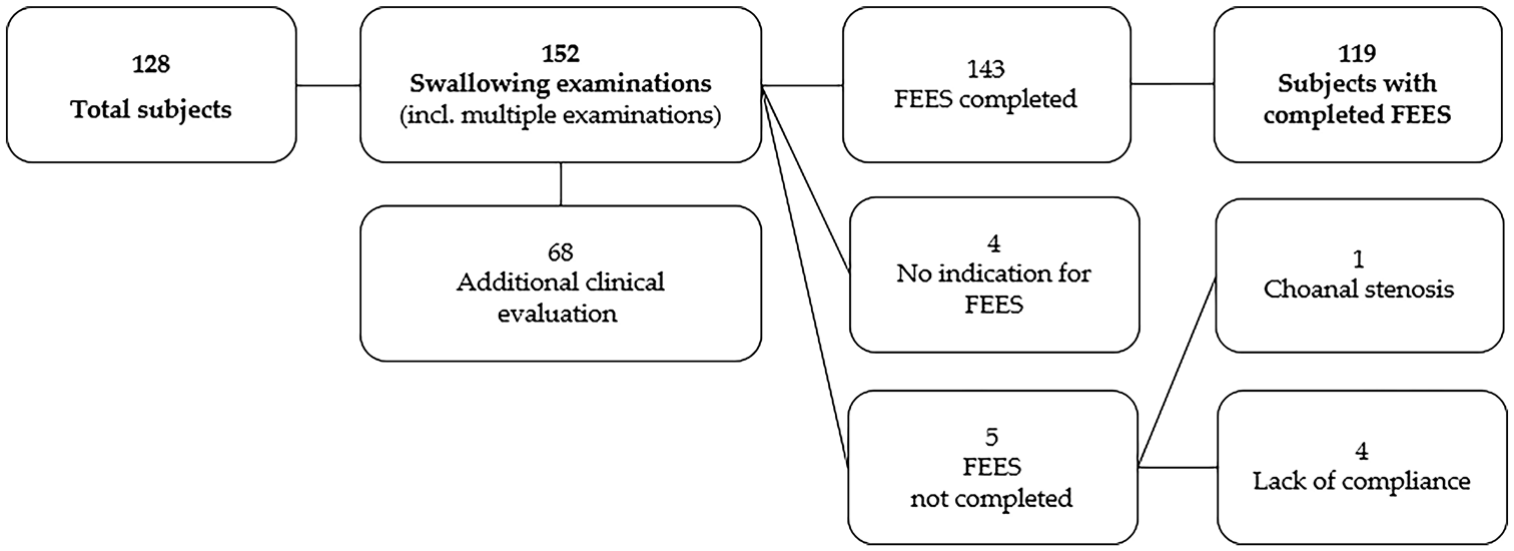
Study population. The diagram shows the number of subjects, the number of conducted examinations, and reasons for discontinued examinations

143 out of 152 pediatric swallowing examinations could be completed as pediatric FEES. In four cases FEES was not performed due to lack of indication. In five cases the examination was discontinued due to lack of compliance (*n* = 4) or the presence of choanal stenosis (*n* = 1) (see Fig. 2).

FEES could be performed in children of all ages. Even in the neo-intensive care bed and in patients under monitoring and/or with an inserted nasogastric tube, the examination could be carried out well. No complications (e.g., epistaxis, apnea) appeared.

The number of examinations increased continuously from 9 in 2015 up to 42 in 2019. In the first 6 months of 2020, 26 children have already been assessed despite a lockdown due to COVID-19.

The gender distribution of children with dysphagia was almost equal (see Table 3). Aspiration was recorded in eight girls and eight boys. In the 15 children with **s**uspected behavioral feeding disorders, the male gender was more frequently affected (3:1).

**Table 3:** Diagnosis (*N* = 128)

	*n* (%)	Male (%)	Female (%)
Dysphagia	69 (53.9)	33 (47.8)	36 (52.2)
Oropharyngeal dysphagia	61 (47.3)	29 (47.5)	32 (52.5)
Dysfunctional sucking	2 (1.6)	0	2 (100)
Suspected esophageal dysphagia	6 (4.7)	4 (66.7)	2 (33.3)
No dysphagia	59 (46.1)	37 (62.7)	22 (37.3)
Suspected behavioral feeding disorder	15 (11.7)	11 (73.3)	4 (26.7)

PAS value was documented in 31 examinations (media* n* = 1, range 1–8). The highest percentage of missing data was found for *laryngopharyngeal sensation* and *delayed swallowing reflex* (see Table 4).

**Table 4:** Swallowing pathologies (*N* = 119^a^)

	Yes (%)	No (%)	Missing (%)
Aspiration	16 (13.4)	80 (67.2)	23 (19.3)
Silent aspiration	3 (18.7)	1 (6.3)	12 (75.0)
Laryngeal penetration (alone)	11 (9.2)	69 (58.0)	28 (23.5)
Spillage	26 (21.8)	43 (36.1)	50 (42.0)
Pharyngeal residue	42 (35.3)	40 (33.6)	37 (31.1)
Delayed swallowing reflex	22 (18.5)	5 (4.2)	92 (77.3)
Decreased laryngopharyngeal sensation	6 (5.0)	6 (5.0)	107(89.9)

^a^Based on 119 subjects with completed FEES

### Impact of Underlying Diseases on Dysphagia

Logistic regression shows that an underlying disease significantly increases the chance of having a positive dysphagia finding (OR 13.08, 95% CI 3.66 to 46.65, *p* = 0.00). More precisely the categories *genetic syndrome* (OR 2.60, 95% CI 1.15 to 5.88) and *neurologic disorder* (OR 4.23, 95% CI 1.31 to 13.69) were associated with a higher chance of dysphagia. In children, without any known disease (*n* = 25) dysphagia was found in only three cases (see Table 5).

**Table 5:** Association between underlying diseases and dysphagia (*N* = 128)

	Dysphagia		Logistic regression			*P* Value^d^
	Yes	No	*β*^a^ (SE)	OR^b^	95% CI	
Underlying disease (*n* = 103)	66	37	2.57 ^b^ (0.64)	13.08^*^	3.66–46.65	.00*
Prematurity (*n* = 14)	7	7	0.64^c^ (0.55)	1.89	.637–5.65	.25
Genetic syndrome (*n* = 43)	27	16	0.95^c^ (0.41)	2.60	1.15–5.88	.02*
Anatomical deviations (4)	3	1	1.58^c^ (1.18)	4.89	0.47–50.31	.18
Neurologic disorder (18)	13	5	1.44^c^ (0.59)	4.23	1.31–13.69	.01*

^a^Regression coefficient

^b^Odds ratio

^c^Cox & Snell *R*^2^ = 0.07. Nagelkerkes *R*^2^ = 0.10. Model *χ *^2^(4) = 10.07. *p* = 0.03

^d^Cox & Snell *R*^2^ = 0.17. Nagelkerkes *R*^2^ = 0.22. Model *χ *^2^(1) = 23.80. *p* = 0.00. *p < .05

## Discussion

### Clinical Observations

In our study, FEES could be performed in children without complications with a completion rate of 96.7%. This decent value can certainly be attributed to the experience of the examiners, as well as the thin diameter of the endoscope, and the consequent use of topical nasal anesthesia [31]. In agreement with Miller and Willging [22], FEES can be confirmed as a feasible and safe procedure for infants, children, and adolescents.

Of the 128 examined children with a suspected swallowing disorder, 54% actually suffered from dysphagia. Aspiration was found in 13.5% of the cohort. In most cases, adequate clearing was performed spontaneously. Only 18.7% of aspirations were silent. This is a moderate rate compared to other studies [20]. It is worth noting that the diagnosis of *no dysphagia* was referred to as absence of pharyngeal swallowing pathologies in FEES. The examiners stated that among the children without pharyngeal pathologies there were definitely children with an oral swallowing disorder.

Logistic regression showed that preexisting or chronic diseases implicate higher odds for children to suffer from pharyngeal dysphagia. Due to the specialization of the cooperating university children´s hospital, the examined cohort comprised a large number of children with genetic syndromes (*N* = 43), including children with rare diseases.

Certainly, this comparatively high proportion of children with a severe or syndromic underlying disease and already existing suspicion of dysphagia leads to a preselection in our cohort. This bias is also evident in other studies that include, for example, a large number of preterm [32] or post-heart surgery patients [33]. Valid data on the dysphagia prevalence in these patient cohorts do currently not exist and only a few studies on endoscopic dysphagia diagnostics have been published. For a better understanding of the swallowing disorders in these children, valid studies are still urgently needed.

Overall, there has been a continuously increasing demand for interdisciplinary swallowing examinations, which has led to an increase in cases of pediatric dysphagia over the past five years.

### Challenges and Future Perspectives

Although a standard FEES protocol has been described for adult patients, [28] and is well established in our university dysphagia center, there is currently no standard procedure for pediatric FEES. This analysis shows that a lack of standard protocol in pediatric FEES causes poor documentation and thus missing values. Probably, if not documented, pathological parameters of the swallowing act such as aspiration and penetration did not occur. This leads to the bias, that the distinction between *absence of pathology* and *not tested* cannot be clearly made in this way. Concerning the less frequently documented findings as *delayed swallowing reflex* and *laryngopharyngeal sensation*, it can be supposed that these were not always routinely examined and documented and could therefore be incorrectly interpreted as a lack of pathology.

Interestingly, a classification of the findings using a rating scale such as the PAS was only carried out in 31 of the analyzed examination documentations. This can be explained by the fact that PAS is well established in adult FEES but cannot simply be transferred to children. This underlines the need for a universally validated assessment standard in children.

Standardized documentation of the pharyngeal pathologies should at least include the *presence*, the *absence,* or the statement *not tested/not assessable* for the relevant items. From our point of view, the findings *early spillover*, *delayed swallowing reflex*, *penetration*, *aspiration*, *clearing*, *residue* and *laryngopharyngeal sensation* would be recommended. To form the basis for improved interdisciplinary communication and treatment in the future, the effect of compensatory strategies (e.g., positioning, pacing, feeding advice) on these pathologies and the resulting dietary (e.g., thickening fluids) and therapeutic recommendations (e.g., gastric tubes) should be documented as well.

Prospectively, the complete pediatric FEES protocol needs to be standardized with necessary variations regarding individual factors such as age/development status, general condition, utilized materials (endoscope, nasal decongestant, local anesthesia, kind of food dye, and thickener), or nutritional modes. Although modified protocols for pediatric FEES are currently being published [21], they still show gaps concerning the entire spectrum of pediatric patients and leave a lot of space for interpretation.

Another task that should not be neglected will be the standardization of CSE. Our analysis shows a high degree of variability in our CSE implementation. Significant gaps in documentation were identified. Similar inaccuracies are also apparent in other studies. As recently reported by Garand et al. [20], there is a great need for specific guidelines even in CSE.

To address these problems in the future, standardization of the entire diagnostic process of pediatric dysphagia is intended in our university dysphagia center as part of the CIDD-P project (clinical and instrumental dysphagia diagnostic standard—pediatric). This will, on the one hand, ensure the best possible care and on the other hand better comparability of studies on epidemiology, evaluation, and treatment outcome in pediatric patients with dysphagia.

## Conclusion

This study shows that FEES in children is well feasible. It also indicates that dysphagia is significantly increased in children with an underlying disease, particularly in genetic syndromes. Despite years of experience in FEES, some deficits in documentation could still be found, which complicates the subsequent scientific processing of data and therefore do not allow for an adequate follow-up. Increased standardization in pediatric FEES is needed. This enables better comparability of studies on epidemiology, assessment, and treatment outcomes of dysphagia in children in the future.

## Supplementary Information

Below is the link to the electronic supplementary material.

Video 1 Excerpts from a FEES recording of an adolescent (14 years old), swallowing thickened liquid, liquid and solid. (AVI 10032 kb)

Video 2 Excerpts from a FEES recording of an infant (24 months), drinking milk from a bottle and eating fruit puree. (AVI 17957 kb)

Video 3 Excerpts from a FEES recording of a child (6,5 years), swallowing yogurt and liquid. (AVI 14548 kb)

Video 4 Excerpt from a FEES recording of a child (6,2 years, nasogastric tube in place), swallowing a small amount of liquid. (AVI 7566 kb)
